# SOX2 Is Regulated Differently from NANOG and OCT4 in Human Embryonic Stem Cells during Early Differentiation Initiated with Sodium Butyrate

**DOI:** 10.1155/2014/298163

**Published:** 2014-02-19

**Authors:** Ade Kallas, Martin Pook, Annika Trei, Toivo Maimets

**Affiliations:** Institute of Molecular and Cell Biology, University of Tartu, Riia 23, 51010 Tartu, Estonia

## Abstract

Transcription factors NANOG, OCT4, and SOX2 regulate self-renewal and pluripotency in human embryonic stem (hES) cells; however, their expression profiles during early differentiation of hES cells are unclear. In this study, we used multiparameter flow cytometric assay to detect all three transcription factors (NANOG, OCT4, and SOX2) simultaneously at single cell level and monitored the changes in their expression during early differentiation towards endodermal lineage (induced by sodium butyrate). We observed at least four distinct populations of hES cells, characterized by specific expression patterns of NANOG, OCT4, and SOX2 and differentiation markers. Our results show that a single cell can express both differentiation and pluripotency markers at the same time, indicating a gradual mode of developmental transition in these cells. Notably, distinct regulation of SOX2 during early differentiation events was detected, highlighting the potential importance of this transcription factor for self-renewal of hES cells during differentiation.

## 1. Introduction

The differentiation potential of human embryonic stem (hES) cells and human induced pluripotent stem (hiPS) cells is a subject of great interest in basic and clinical research. Its investigation will lead to a better understanding of pluripotency and facilitate disease modelling, potential treatment of different pathological conditions, and *in vitro* testing of therapeutic interventions. One of the areas considered to be potentially the most valuable comprises development of protocols for induction of endodermal cells from hES and hiPS cells by using various growth factors (activin A, BMP4, bFGF, EGF, and VEGF) and small molecules (e.g., sodium butyrate, which inhibits histone deacetylases (HDACs) and induces hyperacetylation of histone) [1–10]. Definitive endoderm (DE) is a potential source for generation of endocrine cells like pancreatic cells (beta cells) and hepatic cells such as hepatocytes. Despite the progress in procedures that promote differentiation towards endoderm (and other lineages), there remains a major gap in our understanding of the process of differentiation towards the final cell fate.

Pluripotency of hES cells is maintained by a transcriptional network that is coordinated by the core transcription factors SOX2, OCT4, and NANOG. During differentiation, the levels of these transcription factors are modulated through mechanisms involving epigenetic modifications. Small changes in the level of OCT4 can force pluripotent stem cells to differentiate into cells that express markers of endoderm, mesoderm, or extraembryonic lineages such as trophectoderm-like cells [11, 12]. Similarly, knock-down of SOX2 in hES cells promotes differentiation into trophectoderm-like cells [13], while overexpression of SOX2 induces differentiation to trophectoderm [14]. It is currently unclear how hES cells maintain the expression of these key transcription factors within the narrow limits that permit continuation of the undifferentiated state. In order to begin investigating this, we undertook an analysis of expression of NANOG, OCT4, and SOX2 at the single cell level at pluripotency and during induced differentiation or commitment.

In order to characterize the expression of NANOG, OCT4, and SOX2 simultaneously in individual cells during early differentiation towards endodermal lineage, we used multiparameter flow cytometric method. At the beginning of differentiation, high levels of NANOG, OCT4, and SOX2 were detected in hES cells. However, as differentiation progressed, the levels of OCT4 and NANOG expression decreased, while SOX2 expression was maintained at a high level. The differentiation markers specific to early differentiation into endodermal lineage were first detectable in a hES cell subpopulation coexpressing pluripotency markers NANOG, OCT4, and SOX2 and later in cells expressing SOX2 but not NANOG and OCT4. High expression levels of SOX2 in differentiating cells indicated the importance of this transcription factor to self-renewal and to differentiation towards endodermal lineage. Simultaneous expression of both pluripotency markers and differentiation markers in a single cell demonstrated the gradual mode of developmental transition.

## 2. Materials and Methods

### 2.1. Ethics Statement

This study was conducted using a commercially available human embryonic stem cell line (WA09-H9, National Stem Cell Bank, Madison, WI, USA); no *in vivo* experiments on animals or humans were performed and therefore approval from an ethics committee was not necessary.

### 2.2. Cell Culture

Human ES cell line H9 (WA09, National Stem Cell Bank, Madison, WI, USA) was maintained on Matrigel (BD Biosciences, San Jose, CA, USA) coated plates in mTeSR1 maintenance medium (STEMCELL Technologies Inc., Vancouver, Canada) according to the manufacturer's specifications. The medium was changed daily. After 3-4 days of growth, colonies were detached mechanically with a micropipette tip. After breaking the colonies by gentle pipetting, individual hES cell clumps were plated onto fresh Matrigel coated plates. In order to initiate differentiation, cells with confluence levels of approximately 60–70% (3-4 days after passage) on Matrigel were treated with sodium butyrate (1 mM in RPMI 1640 medium containing 1xB27, both from Invitrogen, Paisley, UK). After 24 h, the medium was replaced with fresh RPMI 1640 (with 1xB27) containing 0.5 mM sodium butyrate, and cells were cultured for further 24–72 h with daily medium changes.

Human embryonal carcinoma-derived (hEC) cell line 2102Ep (GlobalStem, USA) was maintained in DMEM medium (PAA Laboratories, Linz, Austria) containing 10% fetal bovine serum (PAA Laboratories) and MEM Non-Essential Amino-Acids Solution (1 : 100, Invitrogen, USA).

### 2.3. Antibodies and Reagents

Anti-NANOG (PE conjugate), anti-OCT4 (Alexa 647 conjugate), anti-SSEA-4 (stage specific embryonic antigen, Alexa-647 conjugate), anti-SSEA-3 (Alexa-488 conjugate), anti-SOX2 (PerCp-Cy5.5 conjugate) antibodies, and their isotype control antibodies were purchased from BD Biosciences. Anti-GATA4, anti-GATA6, anti-SOX17, anti-SOX9, and anti-FOXA2 antibodies were purchased from Aviva Systems Biology (San Diego, CA, USA). Anti-SOX2 antibody (against C-terminus of SOX2) was obtained from Abcam (USA). Sodium butyrate (Sigma-Aldrich Chemicals, St. Louis, MO, USA) was dissolved and diluted in MQ water.

### 2.4. Multivariate Permeabilised-Cell Flow Cytometry and Cell Cycle Analysis

After harvesting hES cells with 0.05% trypsin-EDTA solution (PAA Laboratories, Linz, Austria) and washing with PBS, single hES cell suspensions were fixed by using 1.6% paraformaldehyde (PFA, Sigma-Aldrich) for 10 min at RT as described for detection of intracellular phosphoproteins [15, 16]. Cells were then washed and stained using a permeabilisation buffer (Foxp3 Staining Buffer Set, e-Biosciences). Cells were blocked using 2% goat serum (LabAs Ltd., Tartu, Estonia) in a permeabilisation buffer (15 min at RT) and stained with appropriate antibodies or their isotype control antibodies for 30 min at RT. For cell cycle analysis, cells were stained with DAPI (Cystain DNA, Partec GmbH, Münster, Germany). Flow cytometry data were acquired with FACSAria using FACSDiva software (BD Biosciences). In some experiments, after fixation with 1.6% PFA, cells were permeabilized with ice-cold methanol for 20 min at 4°C, washed with PBS containing 1% BSA and 2 mM EDTA, and then blocked and stained with antibodies as described above. Cell permeabilisation, fixation and staining, and data acquisition for all samples were done on the same day. The populations positive or negative for specific markers were selected on density plots according to a population's borders or by using specific isotype controls.

### 2.5. Western Blot Analysis

Protein samples were electrophoresed on SDS polyacrylamide gel (10%) and transblotted (MiniTransblot Cell, Bio-Rad, Hercules, CA, USA) onto a polyvinylidene difluoride membrane (Millipore, Billerica, MA, USA). The membranes were probed with rabbit anti-NANOG, anti-SOX17, anti-SOX9, anti-GATA4, anti-GATA6, and anti-FOXA2 antibodies (Aviva Systems Biology), mouse anti-OCT4 (Santa Cruz Biotechnology, San Diego, CA, USA), and rabbit anti-SOX2 antibodies (Abcam, Cambridge, MA, USA) followed by horseradish peroxidase-conjugated goat anti-rabbit or goat anti-mouse secondary antibodies. Mouse anti-beta-actin antibody (Abcam) was used for detecting the loading control. Binding of antibodies was visualized with ECL reagent (Western Lightning Plus-ECL, PerkinElmer Inc, Waltham, MA, USA) and exposing the blots on X-ray films (Amersham Biosciences).

### 2.6. Statistical Analysis

A two-tailed paired *t*-test with a confidence interval of 95% was used to analyse the data with GraphPad Prism 4 software. *P* value less than 0.05 was considered significant. All results are presented as mean ± standard error.

## 3. Results

### 3.1. The Expression Pattern of Pluripotency Markers NANOG and OCT4 Is Different from That of SOX2 in Differentiating hES Cells

**Figure 1:**
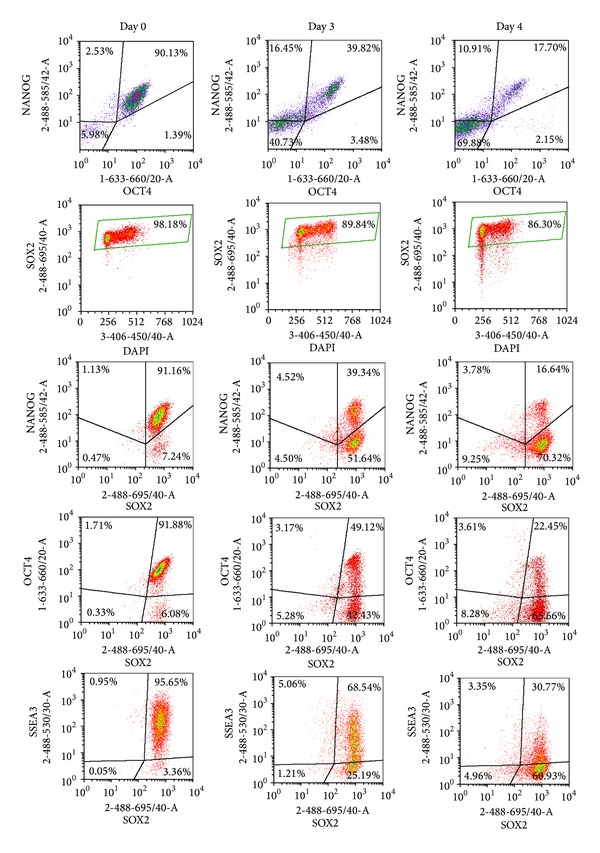
Changes in the expression patterns of pluripotency markers NANOG and OCT4 are distinct from those of SOX2 in differentiating H9 cells. hES cells were treated with sodium butyrate as described in Section 2. Coexpression of pluripotency markers during differentiation on days 3 and 4 was detected by flow cytometry. Fixed and permeabilised cells were stained with anti-SSEA-3 (Alexa Fluor 488 conjugate), anti-NANOG (PE), anti-OCT4 (Alexa Fluor 647), and anti-SOX2 (PerCp Cy5.5 conjugate) antibodies as well as with DAPI. Cellular debris and doublets were excluded from analysis. Results are representative of two independent experiments.

Firstly, we assessed the coexpression of transcription factors NANOG, OCT4, and SOX2 in pluripotent hES cells (Figure 1, day 0). Most pluripotent hES cells coexpressed high levels of NANOG and OCT4 (90% NANOG+OCT4+ cells). The number of SOX2 expressing cells was even higher (98% SOX2+ cells), and most of these also expressed SSEA-3 (95% SOX2+SSEA3+ cells). The coexpression of NANOG, OCT4, and SOX2 was detected in 91% of cells, demonstrating that during regular culture of hESC a small subpopulation (usually less than 10%) of cells expressed SOX2 and SSEA-3, but not NANOG and OCT4. SOX family members possess a high degree of homology, particularly in their DNA binding domains. Therefore, we confirmed our results using another SOX2-specific antibody that detects the C-terminal part of the protein. As seen in Supplementary Figure 1 (Supplementary Materials available online at http://dx.doi.org/10.1155/2014/298163), both antibodies used in this study detected SOX2 with similar efficiencies.

Next, we asked whether the expression of SOX2 and other transcription markers changes during cell differentiation towards endoderm induced by sodium butyrate [3, 17–19]. We used a differentiation protocol in which the cells were grown on Matrigel coated plates in mTeSR1 medium for 3-4 days, and thereafter in differentiation medium containing sodium butyrate for further 3-4 days. We applied the first step of the differentiation protocol used for initiation of functional hepatocyte-like cells [20]. The number of cells coexpressing OCT4 and NANOG decreased significantly (39% and 17% of NANOG+OCT4+ cells by days 3 and 4, resp., Figure 1). The number of cells expressing SOX2 decreased somewhat from 98% at the beginning of differentiation to 86% by day 4 (Figure 1). Nevertheless, expression levels of SOX2 were high and there was no significant difference in the levels of SOX2 in NANOG+OCT4+SOX2+ cells with mean fluorescence value (MFI) of 735 compared to NANOG−OCT4−SOX2+ cells with MFI value of 756. The number of SSEA-3 expressing cells decreased rapidly from 95% to 30% by day 4, indicating changes taking place on the cell surface (Figure 1). The morphology of cell colonies changed from compact to less organized and smaller structures (Figure 4(d)).

To find out whether the changes in transcription factor expression induced by sodium butyrate treatment were reversible, we treated the cells with sodium butyrate (1 mM) in differentiation medium for 24 h and then, after careful washing, the cells were grown in mTeSR1 medium for further 24 h. A decrease in NANOG and OCT4 coexpressing cells was detected after sodium butyrate treatment (from 91% to 67%). The population of cells without NANOG and OCT4 coexpression increased (from 6% to 29%) and these cells expressed SOX2 (Supplementary Figure 2). Thus, nearly all cells continued to express SOX2. After removal of sodium butyrate, 84–86% of cells expressed all three transcription factors, while 11–14% of cells expressed SOX2, but not NANOG or OCT4. These observations indicate that the effects of sodium butyrate treatment on expression of key pluripotency markers are largely reversible.

The results obtained by analyzing hES cells indicate that expression of SSEA-3, NANOG, and OCT4 is very sensitive to treatment with sodium butyrate, while SOX2 is regulated by different mechanisms. In contrast, when human embryonal carcinoma-derived (hEC) 2102Ep cells were treated with sodium butyrate, they continued to express high levels of SSEA-3, SSEA-4, NANOG, OCT4, and SOX2 (Supplementary Figure 3). As no changes in transcription factors expression were detected in hEC cells, only hES cells were used in subsequent experiments.

### 3.2. Expression of Differentiation Markers in hES Cells

**Figure 2:**
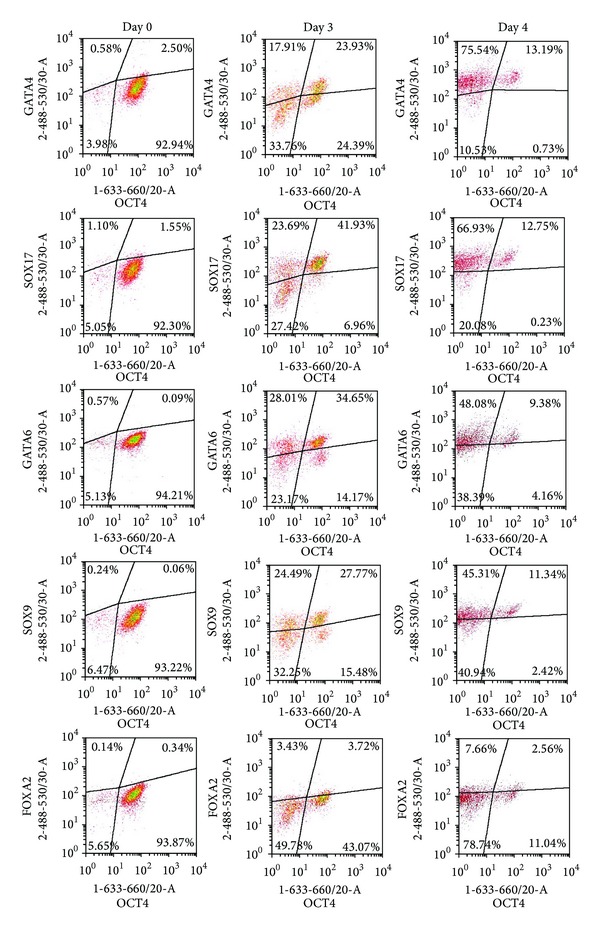
Detection of pluripotency marker OCT4 and differentiation markers in differentiating H9 cells. hESC were treated with sodium butyrate as described in Section 2. Cells harvested before initiation of differentiation (day 0) and 3 or 4 days later were fixed with 1.6% PFA and permeabilised with ice-cold methanol. Coexpression of pluripotency marker OCT4 and differentiation markers on days 3 and 4 was detected by flow cytometry.

**Figure 3:**
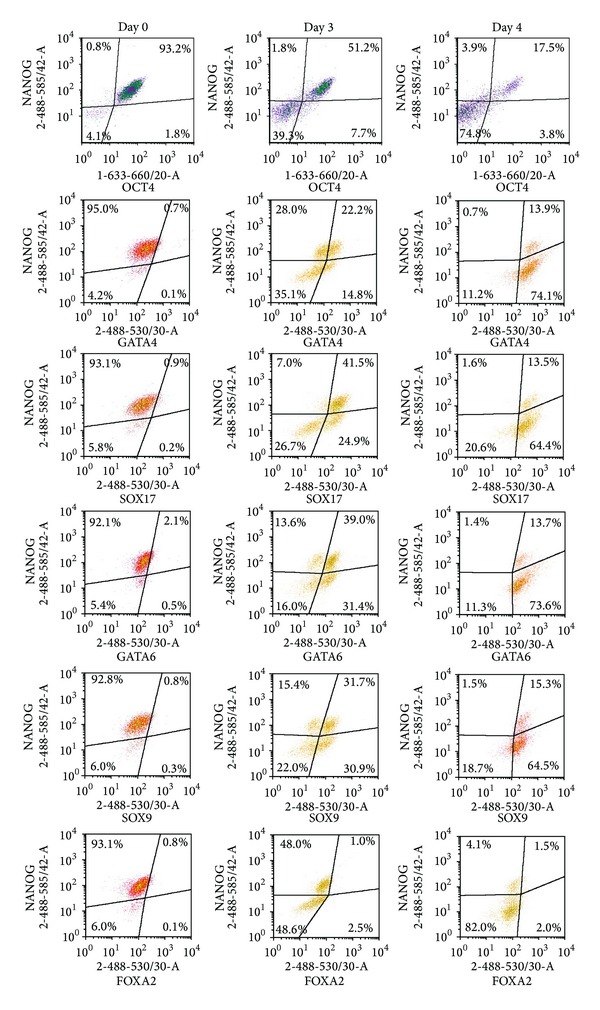
Expression of pluripotency marker NANOG and differentiation markers in differentiating H9 cells. Cells were treated and processed as described in the legend of Figure 2. Coexpression of pluripotency markers NANOG and OCT4 or of NANOG and differentiation markers on days 3 and 4 was detected by flow cytometry.

**Figure 4:**
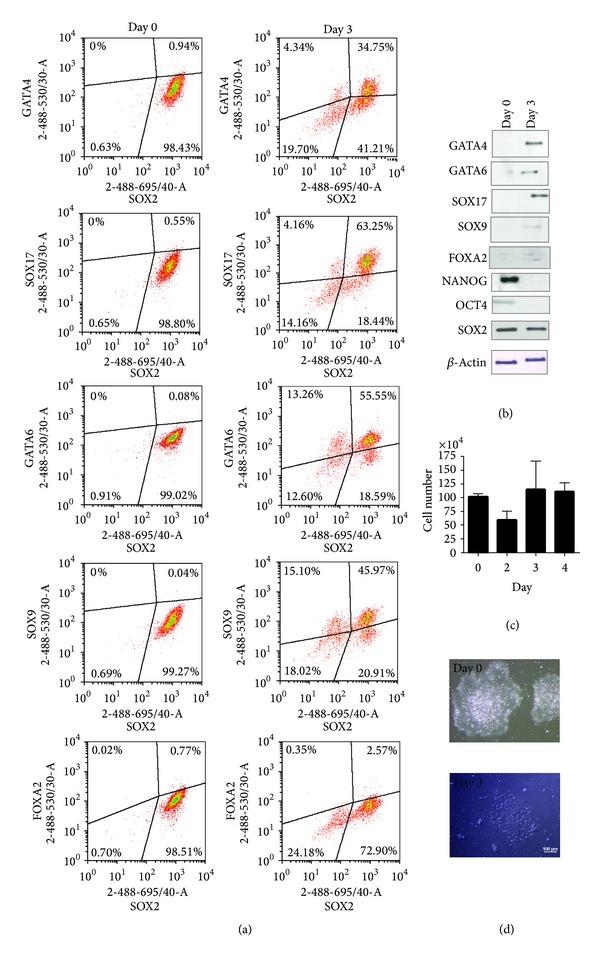
Detection of pluripotency marker SOX2 and differentiation markers in differentiating H9 cells. Cells were treated and processed as described in the legend of Figure 2. Coexpression of pluripotency marker SOX2 and differentiation markers was detected by flow cytometry (a) or by Western blot (b). Results are representative of two independent experiments. (c) The number of cells in one well is shown as a mean value ± SEM of three experiments. (d) The changes in colony morphology detected at the beginning of differentiation (day 0) and on day 3.

Next, we investigated whether the expression of differentiation markers could be detected at early stage of differentiation. To carry out flow cytometric assays, we optimized the cell treatment protocol by using 1.6% PFA for fixation and ice-cold methanol for cellular permeabilisation. This procedure resulted in lower nonspecific background fluorescence signal, as well as appropriate detection of positive and negative cell populations by flow cytometry. In addition, this modified protocol allowed us to use antibodies suitable for Western blotting, whose epitopes are more linear and denatured than those used in flow cytometric assays. In our set-up, we used differentiation markers GATA4, GATA6, SOX17, SOX9, and FOXA2. Except FOXA2, all markers are detectable at the early stages of differentiation into endodermal lineage, notably into visceral and definitive endoderm [21]. Sodium butyrate treatment initiated differentiation of cells: distinct populations of GATA4, GATA6, SOX17, and SOX9 producing cells were detectable by day 3 (Figure 2). At this stage of differentiation, large numbers of cells coexpressed NANOG, OCT4, and SOX2. Thus, we tested hES cells for simultaneous coexpression of a single differentiation marker and a single pluripotency marker (Figure 2). By day 3, we could detect four distinct subpopulations of cells according to the expression of OCT4 and the differentiation marker GATA4: (1) OCT4+GATA4+, (2) OCT4+GATA4−, (3) OCT4−GATA4+, and (4) OCT4−GATA4−. It was interesting to note that GATA4 expression was detected both in OCT4 expressing cells and in a subpopulation of cells where OCT4 expression was downregulated. Similar distribution of subpopulations was found when analysing expression of GATA6, SOX9, and SOX17. Only FOXA2 expression was barely detectable by day 3, but Western blot analysis confirmed its presence in differentiating cells (Figure 4(b)). By day 4, expression of differentiation markers increased and almost all OCT4 expressing cells also expressed GATA4, GATA6, SOX17, and SOX9 (Figure 2). Figure 2 demonstrates expression of OCT4, while Figure 3 shows that the pattern of NANOG expression was similar. Since cells that expressed OCT4 also expressed NANOG and no distinct subpopulations of cells expressing only NANOG or OCT4 were detectable, we refer to these coexpressing cells as NANOG/OCT4 double positive cells (Figure 3).

Since SOX2 expression during differentiation differed from that of NANOG and OCT4, we asked next whether differentiation markers could be detected in cells expressing SOX2. We found that the majority of cells expressing GATA4 or SOX17 expressed SOX2 by day 3, while only 4% of cells expressing GATA4 or SOX17 did not express SOX2. Similarly, only 13% and 15% of GATA6 or SOX9 expressing cells, respectively, were negative for SOX2 expression (Figure 4(a)). The cells without SOX2 expression were found to be mostly in the G1 phase, indicating the gradual changes in the cell cycle during differentiation (Figure 1). These findings indicate that SOX2 may be involved in cellular proliferation and self-renewal of hES cells during early differentiation.

**Figure 5:**
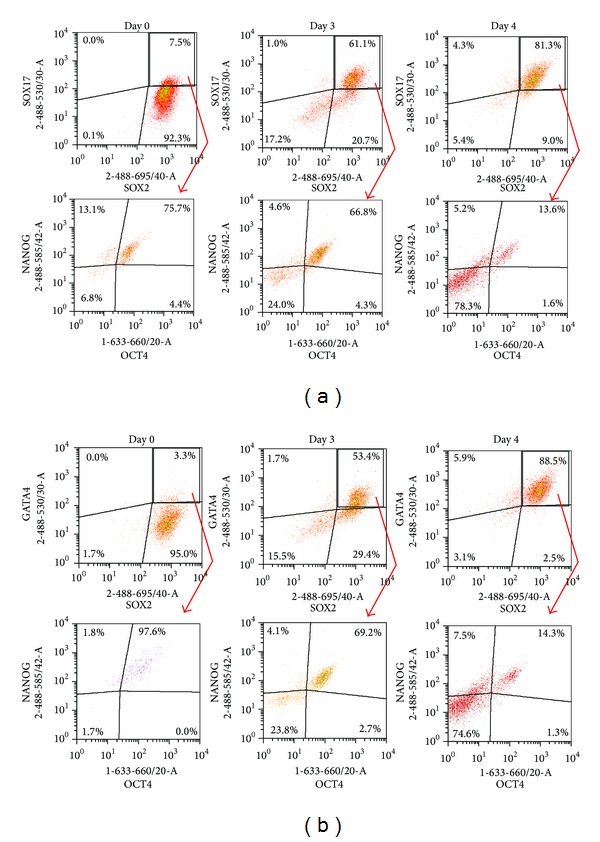
Characterisation of SOX17 and GATA4 expressing cells for expression of pluripotency markers NANOG, OCT4, and SOX2. Cells coexpressing SOX17 (a) or GATA-4 (b) and SOX2 were selected and coexpression of NANOG and OCT4 in these cells was estimated.

Recently, it has been demonstrated that OCT4 can form a dimer with another member of the SOX family of proteins, SOX17, which mediates differentiation of hES cells [22]. It is possible that the ratio of OCT4 to SOX2 and to SOX17 expression determines whether cells remain pluripotent or initiate the differentiation process [11]. For more detailed analysis of cells coexpressing differentiation and pluripotency markers, we selected the subpopulation coexpressing SOX17 and SOX2 on a density plot (Figure 5(a)) and analyzed it for expression of NANOG and OCT4. At the beginning of differentiation, only 7.5% of cells were SOX17+SOX2+, and 75% of these coexpressed NANOG and OCT4 (Figure 5(a)). By day 4, 81% of cells expressed SOX17 and SOX2, and 14% of this subset were NANOG and OCT4 positive. Similar trends were observed when GATA4+SOX2+ cells were examined (Figure 5(b)). Therefore, we conclude that during early stages of differentiation, the increase in SOX17 expression is accompanied by a decreased expression of NANOG and OCT4. In addition, these results also support our finding that expression of NANOG and OCT4 is regulated similarly, whereas regulation of SOX2 follows a different pattern.

## 4. Discussion

Artificially induced differentiation of stem cells is a promising tool for generating various cell types and tissues for therapy of different disorders. Therefore, it is highly important to obtain detailed information about the cellular processes that take place during differentiation. In this study, we characterized at single cell resolution the changes in levels of transcription factors responsible for pluripotency. In individual hES cells treated with sodium butyrate to initiate differentiation, we could simultaneously detect early differentiation markers GATA4, GATA6, SOX17, and SOX9, as well as pluripotency markers NANOG, OCT4, and SOX2. This finding demonstrates the gradual mode of developmental transition in these cells.

We detected expression of early differentiation markers GATA4, GATA6, SOX17, and SOX9 as early as day 3 of differentiation. Some of the differentiating cells also coexpressed NANOG, OCT4, and SOX2 at this time point. Our findings are in accordance with previously published results showing coexpression of early differentiation markers and pluripotency markers in hES cells [18, 21, 23]. These observations highlight the need to estimate simultaneously the expression of transcription factors NANOG, OCT4, and SOX2 as well as markers for differentiation in hES cells for characterizing pluripotency and quality of cell culture. The differentiation marker FOXA2, which has been reported to be detectable on days 3–6 in the presence of activin A [17, 18, 21], was not detected by flow cytometry and was barely detectable by Western blotting. The fact that sodium butyrate exerts a different effect on hES cells than activin A (activates TGF-*β* signalization pathway and induces modulation of transcription factor complexes [24–26]) may explain the low level of FOXA2 in differentiating cells observed in this study.

Our results show that histone deacetylase activity is required for OCT4, NANOG, and SSEA-3, but not SOX2 expression in hES cells. Inhibition of HDACs by sodium butyrate resulted in expression of differentiation markers GATA4, GATA6, SOX9, and SOX17, while removal of sodium butyrate restored the expression of NANOG and OCT4 in SOX2 expressing cells demonstrating that deacetylation is important for maintaining a pluripotent state and preventing hES cell differentiation. Indeed, OCT4 has been shown to be involved in global acetylation of active chromatin and maintenance of hES cells in a pluripotent state [27]. Recently, it has been shown that differentiation of hESC towards oligodendrocytes by using HDAC inhibitors (trichostatin A, sodium butyrate) had no effect on SOX2 mRNA levels [28]. In this study, we confirmed that SOX2 protein levels were also not affected by sodium butyrate treatment. It is interesting to note that in differentiated cells, sodium butyrate can reprogram cells to pluripotent stem cells via a mechanism mediated by regulation of the miR302/362 cluster [29]. Earlier studies in mouse EC cells (F9 cell line) have shown only morphological changes as a response to sodium butyrate with no effective differentiation [30]. We detected that, in contrast to hES cells, sodium butyrate treated hEC cells still expressed SSEA-3, SSEA-4, and transcription factors NANOG, OCT4, and SOX2 at high levels, suggesting a difference in regulation of transcription factors responsible for pluripotency in hES and hEC cells. Thus, interference with HDAC activity may lead to different outcomes depending on the differentiation status of the cell.

Sodium butyrate in the presence or absence of activin A has been successfully used in protocols of differentiating hES cells into pancreatic cells producing insulin [3] or into functional hepatocyte-like cells expressing hepatocyte markers [18, 20]. In addition, induction of endodermal-specific gene expression was found to be accompanied by upregulation of several liver-enriched miRNAs, including miR-122 and miR-192, as observed in hES cells treated with sodium butyrate [31]. Transient treatment with another HDAC inhibitor, MS-275, induced epigenetic modifications in mouse ES (mES) cells, preventing teratocarcinoma formation [32]. However, the effect of a transient application of MS-275 was found to be reversible, as after its removal and long-term culture of mES cells (more than 4 passages) colony formation ability recovered, as did pluripotency [32]. As we show in this study, removal of sodium butyrate after a transient application restores expression of NANOG and OCT4 in cells expressing SOX2, confirming the reversible nature of HDAC inhibition in hES cells, which has not been described earlier. Thus, a change in culture conditions to those used for culturing pluripotent cells (i.e., presence of basic fibroblast growth factor, etc.) may cause cells expressing SOX2 but not NANOG and OCT4 to revert to expressing all three transcription factors. This finding also highlights the important role played by SOX2 and confirms the different pattern of its regulation compared to NANOG and OCT4.

By using different markers of hES cells pluripotency, we delineated correlations of expression of certain markers, which can be utilised for characterisation of pluripotent hES cells. In untreated hESC, the expression of SSEA-4 is widely acknowledged (most cells express SSEA-4) [33, 34], but as we have shown previously, expression of SSEA-3 correlates more precisely with coexpression of NANOG and OCT4 [33]. Furthermore, by utilising SOX2 detection, two subpopulations of hESC could be characterized: SSEA-3+NANOG+OCT4+SOX2+ cells and SSEA-3+NANOG−OCT4−SOX2+ cells. This finding is in agreement with other reports, where the subpopulation hierarchy was established by starting with the marker with the lowest expression [18, 21, 23] and interpreted as a heterogeneity of hESC. By applying multiparameter flow cytometric analysis instead of analysing only one parameter allowed us to establish a correlation in expression of pluripotency markers. This is a notable finding since, as we show in this study, heterogeneity of cells becomes an important issue during differentiation or any manipulation of hES cells as shown in our previous study [33].

In hESC, OCT4 can act as a dose-dependent switch regulating the transition from pluripotency to induction of cardiogenesis, due to its interaction with the pluripotency marker SOX2 or with the differentiation marker SOX17 [11, 12]. It has been suggested that the level of OCT4 determines whether OCT4 targets the OCT4−SOX2 enhancer, thus maintaining the NANOG, OCT4, and SOX2 expression, or instead binds SOX17 to drive cells towards the endo- or mesodermal lineage [35]. It has been shown that high levels of OCT4 or low levels of SOX2 induce OCT4 binding to the SOX17 promoter but that OCT4 alone is still not capable of directly activating lineage specific genes [35]. Thus, changes in OCT4 levels may guide hES cells towards endoderm formation, which is stimulated further by culture conditions. In this study, a multiparameter flow cytometric method allowed us to detect the changes in expression levels of OCT4, NANOG, SOX2, and SOX17. Indeed, by day 3 of differentiation, we could detect expression of SOX17 in OCT4/NANOG and SOX2 expressing cells. However, as expression of SOX17 (and likely other differentiation markers) increased, expression of NANOG/OCT4 in these cells dropped—by day 4 only 13.6% of SOX2 and SOX17 expressing cells coexpressed NANOG/OCT4 (Figure 5(a)). Although the stoichiometry of OCT4 and SOX2 expression changed, expression of SOX2 remained high during differentiation initiated by sodium butyrate.

High expression of SOX2 in cells differentiating towards endodermal lineage has not been reported so far. In neurogenesis, SOX2 is expressed in progenitor cells, is responsible for cell proliferation [36], and generates neural precursors as well as SOX2+ neural stem cell population [37]. Pancreatic progenitors do not originate from one source as shown by embryogenesis studies [38]. The similarities in development of pancreatic beta cell and neuroepithelial cells have been shown [39], and applying the formation of embryonic body development as a first stage in protocol differentiating cells towards endodermal lineage has been as effective as other protocols [40]. Therefore the finding that SOX2 expression is high in differentiation towards neural or endodermal progenitors could be expected.

By day 4 of differentiation, expression of SOX2 was decreased and approximately 10% of cells lost SOX2 expression. We found that most of these cells were in the G1 phase of the cell cycle. As elongation of the G1 phase and shortening of the S phase are characteristic of the cell cycle changes that take place in differentiated cells, we conclude that these cells were indeed differentiated. Thus, it appears that SOX2 expression may not be crucial in later stages of differentiation. Our finding argues that SOX2 is important for proliferation and self-renewal in addition to being a lineage specific marker in differentiation. Indeed, recent comparisons of the SOX2 interactomes in ES cells before and after the initiation of differentiation have shown that this protein's interactions change dramatically within 24 hours [41]. Less than a third of the SOX2-associated proteins are present in the SOX2 interactomes of both untreated hES cells and of those undergoing differentiation [41]. Our finding that SOX2 is highly expressed in differentiating hES cells as well as in pluripotent cells suggests that this protein may be involved in maintenance of proliferation and self-renewal. It is likely that SOX2 possesses different roles in pluripotent and in differentiating cells: we could detect SOX2 expression in pluripotent cells expressing NANOG/OCT4 as well as in differentiating cells expressing GATA4, GATA6, SOX17, and SOX9. Additionally, it is possible that posttranslational modifications occur in SOX2 and in SOX2 associated proteins. For instance, it has been reported that one hour after initiation of differentiation in hES cells, the phospho-proteome changes by ~50% [42]. Furthermore, in hES cells, transcription factors such as SOX2 and OCT4 undergo not only phosphorylation but also acetylation, poly(ADP-ribosyl)ation, methylation, sumoylation, and glycosylation [43–49]. These changes in posttranslational modifications of interacting proteins are attractive candidates for their regulatory mechanisms, as well as for possible harnessing to experimentally maintain pluripotency or induce efficient differentiation into endoderm. Further analysis of the changes that occur in binding of transcription factors genome-wide as well as in the protein-protein interaction networks during the initial stages of differentiation will provide a better understanding of the molecular events that accompany the loss of hES cell pluripotency.

## 5. Conclusions

The use of human embryonic stem (ES) cells has been attractive for laboratory studies and for cell-based therapies. However, their application is complicated by their high propensity to lose pluripotency, as well as by difficulties in generating pure populations of differentiated cell types *in vitro*. Pluripotent stem cells self-renew indefinitely and possess characteristic protein-protein networks that are remodeled during differentiation. How this occurs is poorly understood. In this study, differentiation of hES cells initiated with sodium butyrate, which inhibits histone deacetylation, showed that a single cell can express both differentiation and pluripotency markers at the same time, indicating the gradual mode of developmental transition in these cells. Unique regulation of transcription factor SOX2 during early differentiation events was detected, suggesting that this protein may be important for self-renewal of hES cells during differentiation. This study also highlights the importance of characterising hES cell cultures for simultaneous expression of pluripotency markers and differentiation markers in a single cell.

## Supplementary Material

Detection of SOX2 by using two different antibodies is shown in Figure 1.
Figure 2 describes expression of pluripotency markers in hES cells after transient treatment with sodium butyrate.
Figure 3 describes distinct patterns of pluripotency marker expression in hES (H9) and hEC (2102Ep) cells after treatment with sodium butyrate.
