# Supplementary material for: SOX2 Is Regulated Differently from NANOG and OCT4 in Human Embryonic Stem Cells during Early Differentiation Initiated with Sodium Butyrate
Source: Stem Cells Int. 2014 Feb 19;2014:298163. doi: 10.1155/2014/298163 (PMC3951062; doi:10.1155/2014/298163)
Supplement: Supplementary file 1 — Detection of SOX2 by using two different antibodies is shown in Figure 1. Figure 2 describes expression of pluripotency markers in hES cells after transient treatment with sodium butyrate. Figure 3 describes distinct patterns of pluripotency marker expression in hES (H9) and hEC (2102Ep) cells after treatment with sodium butyrate. [file 298163.f1.doc]

**Supplementary Materials**

**Supplementary Figure 1.** **Detection of SOX2 by using two different antibodies and co-expression of SOX2 with NANOG and with OCT4 in differentiating hES cells.** (A) Cells (before the differentiation, day 0, and by day 3 after initiation of differentiation) were harvested and fixed with 1.6% PFA and permeabilised with ice-cold methanol and stained with anti-SOX2 (detecting C-terminus of SOX2, Alexa Fluor 488), anti-NANOG (PE), anti-OCT4 (Alexa Fluor 647), anti-SOX2 (detecting full length of SOX2, PerCp Cy5.5) antibodies and DAPI. (B) Detection of SOX2 by two different antibodies recognizing full-length SOX2 or the C-terminus of SOX2.

**Supplementary Figure 2.** **Distinct patterns of expression of pluripotency markers NANOG and OCT4 *versus* SOX2 in hES cells after transient treatment with sodium butyrate.** hES cells were treated with sodium butyrate (1mM) for 24h in differentiation medium (24h sodium butyrate) and again for 24h after wash-out of sodium butyrate in mTeSR^TM^1 medium (WO, 24h medium). (A) Expression of NANOG, OCT4 and SOX2 was detected as described in the legend to Figure 1. (B) The number of cells in one well is shown as a mean value ± SEM of two experiments. (C) Changes in colony morphology detected at the beginning of differentiation (day 0), after 24h treatment with sodium butyrate, and after its removal for 24h.

**Supplementary Figure 3. Distinct patterns of pluripotency marker expression in hES (H9) and hEC (2102Ep) cells after treatment with sodium butyrate.** hES and hEC cells were treated for 24h with sodium butyrate (1mM) in differentiation medium as indicated in Materials and Methods and cells were analysed as described in the legend to Figure 1.

**Supplementary Figure 1**

**
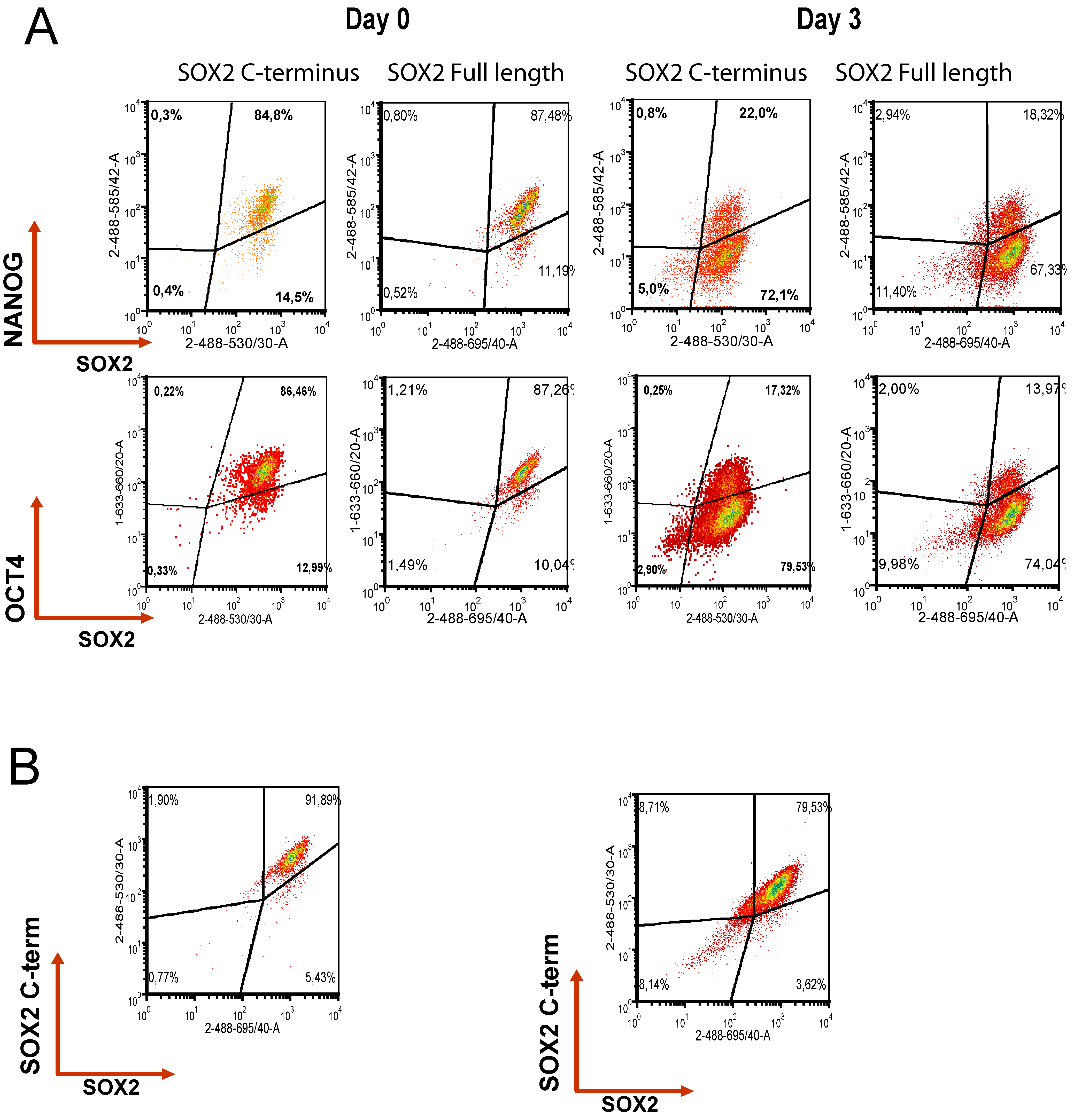
**

**Supplementary Figure 2.**

**
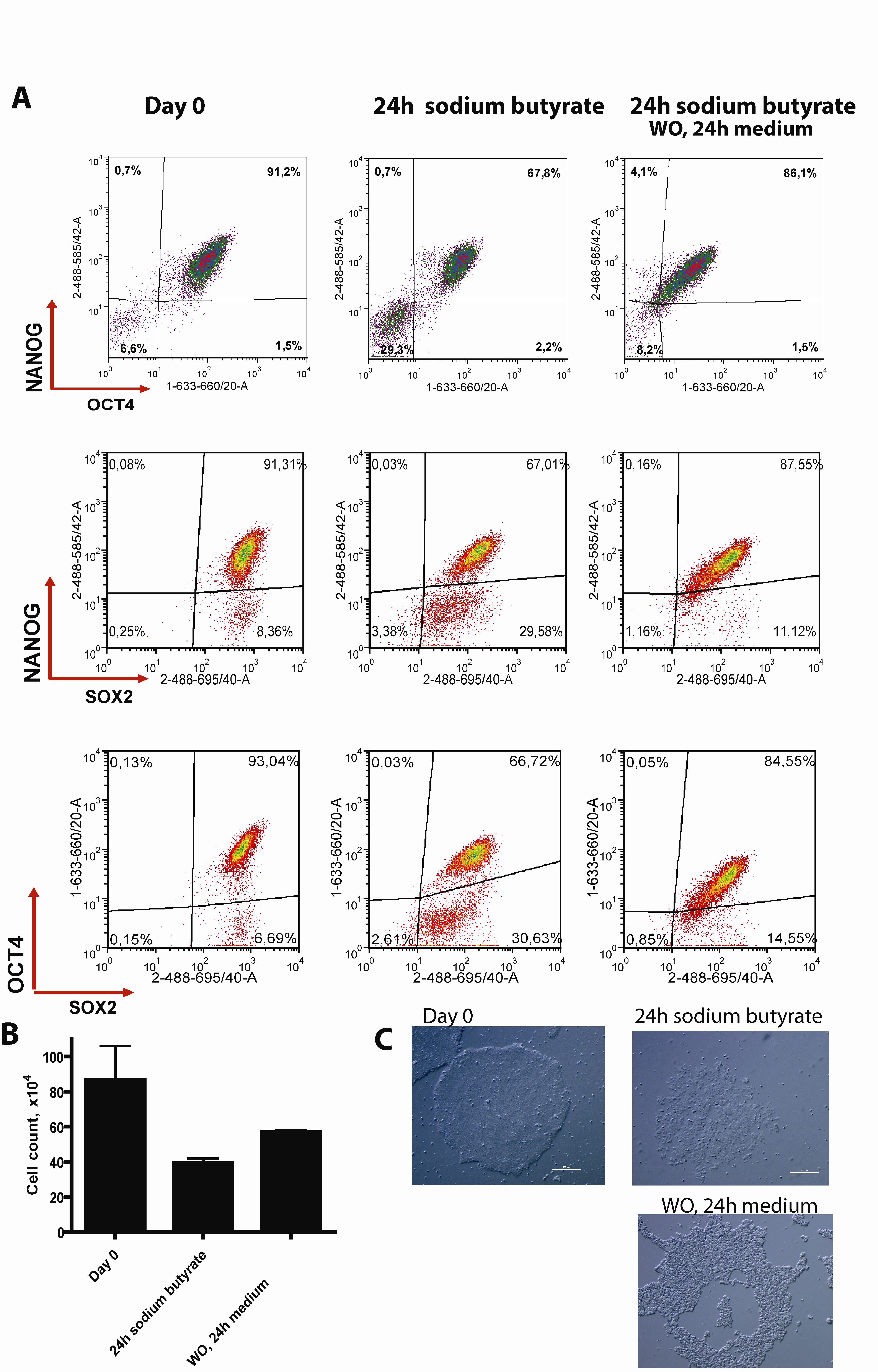
**

**Supplementary Figure 3.**

**
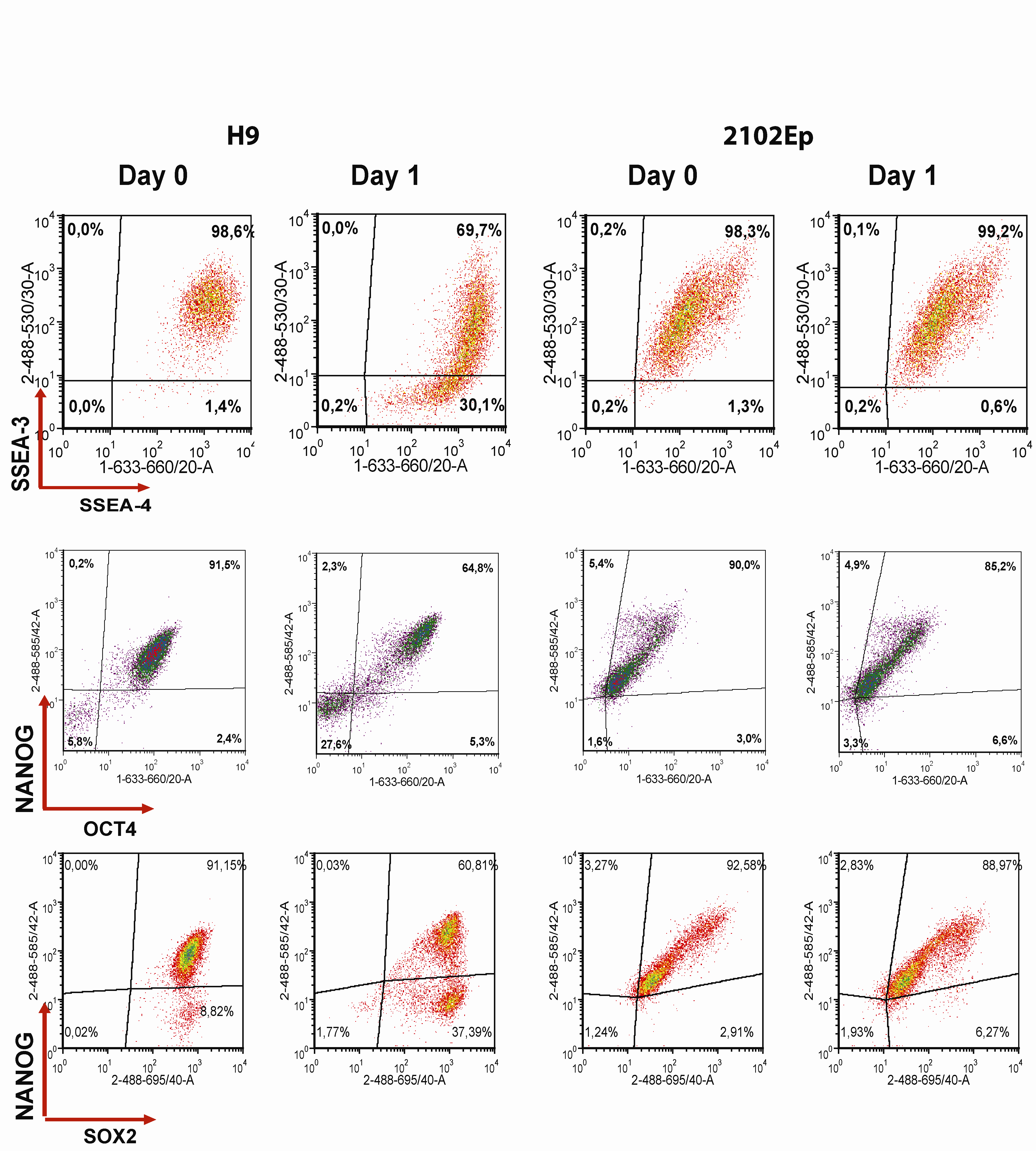
**
